# The Interplay between Maternal Nutrition and Oxidative Stress

**DOI:** 10.3390/nu15092194

**Published:** 2023-05-05

**Authors:** Enrico Maria Ferrazzi

**Affiliations:** 1 Fondazione IRCCS Ca’ Granda Ospedale Maggiore Policlinico, Department of Mother, Child and Neonate, 20122 Milan, MI, Italy; enrico.ferrazzi@unimi.it; 2 Department of Clinical Sciences and Community Health, University of Milan, 20122 Milan, MI, Italy

This Special Issue of *Nutrients*, “The Interplay between Maternal Nutrition and Oxidative Stress”, was designed to contribute to our understanding of “oxidative stress” in pregnancy.

The complex balance between free radicals, such as reactive oxygen species and antioxidant defense mechanisms, is a lifelong challenge that eventually leads to mitochondrial damage, sirtuin dysfunction, chromatin “scratches”, telomere damage, cell senescence, and death. The human placenta is a unique organ with a short lifespan in which this balance is the result of the interaction of the exposome with an individual’s immune, cardiovascular, genetic, and epigenetic profile, leading to its senescence and a proinflammatory condition at the end of its life at 38–40 weeks and shortly beyond.

In the short life of this organ, unbalanced maternal nutrition may have a negative effect, increasing the risk of miscarriage, hypertensive disorders, and pregnancy complications, or a positive effect, supporting antioxidant mechanisms and a healthy pregnancy and newborn.

In this Special Issue, the role of diet in increasing the risk of chronic inflammation in pregnant and non-pregnant adults [1,2] is addressed by a paper that directly measured the impact of the Diet Inflammation Index on the perinatal outcome of a large cohort of at-risk pregnant women [3] included in the IMPACT BCN Trial [4]. This study proved a significant association between an anti-inflammatory diet and a higher newborn weight. 

Pregnant women whose diet had the lowest inflammatory index had a higher intake of antioxidants with their diet. The antioxidants present in fruits, vegetables, extra-virgin olive oil, nuts, and dietary fiber are known to be negatively associated with inflammation. A low inflammatory index was also associated with healthy food sources of animal protein, such as blue fish.

Late fetal growth restriction is a common poor obstetrical outcome that occurs in up to ten percent of pregnancies. Effective preventive lifestyle measures that improve fetal growth in utero, such as those described in this study, have enormous potential in clinical obstetrics.

In this Special Issue, the possible impact of oxidative stress resulting from metabolic syndrome in pregnancy was also investigated by a study that involved a large cohort of pregnant women diagnosed with gestational diabetes [5] in accordance with the HAPO study criteria [6]. These criteria aid in screening for the inappropriate handling of glucose in a standard glucose tolerance test. Other constituents are required to define metabolic syndrome in pregnancy [7], namely, hypertension, dyslipidemia, and proteinuria. These concurrent constituents of metabolic syndrome cumulatively increase the risk of the later development of preeclampsia, altered fetal growth, and preterm birth. Obesity itself did not show any relation to adverse outcomes, but it is of note that this condition was observed in half of the cases of gestational diabetes and in one in four non-affected patients. Additionally, its impact was diluted by both the statistical prevalence of obesity in this cohort and by the treatment paradox. Obviously, these patients were properly treated by diet, metformin, and/or insulin according to a strict criterion based on a longitudinal fetal growth target no higher than the 40th percentile from mid-trimester at the time of glucose testing.

In an environment of low-grade inflammation, the lipid metabolism alterations in gestational diabetes, promoted by the placental tissue, were further investigated by a study reported in this Special Issue on the maternal AA/EPA ratio and triglycerides in pregnant women affected by gestational diabetes [8].

An appropriate diet effectively reduces the production of pro-inflammatory eicosanoids. This approach has been proposed by multiple studies to avoid pharmacological treatment and to reduce reactivity in pregnancy [9]. This study, reported in this Special Issue, compared triglyceride concentrations and AA/EPA ratios in a randomized trial of pregnant patients with gestational diabetes who were allocated to the Mediterranean diet (MD) and to the MD with the inclusion of polyphenols of a plant origin.

The authors’ conclusions are of interest. The main overall result from comparing the two arms of the trial did not demonstrate significant differences; as such, it casts light on the role of nutritional coaching on one side and the efficacy of nutraceutical supplementation on the other: “The effects of supplementation with omega-3 and antioxidants on metabolic and inflammatory parameters of women with GD were not significant compared with strict and one-to-one careful nutritional consultation that paired the effects of supplementation”. The second conclusion stems from this result, which allowed the authors to analyze the cohort altogether and observe that the triglyceride concentration and AA/EPA ratio were biomarkers for higher inflammatory levels and patients with gestational diabetes who were candidates for pharmacological treatment: “An adequate assumption of omega-3 in women with GD, either by a controlled diet or by nutraceutical supplementation, reduces the need for pharmacological therapy and omega-3 fatty acids”.

On the other side of oxidative stress and inflammation in pregnancy are hypertensive disorders (HDPs). However, two main HDP phenotypes have been proposed: on one side are HDPs resulting from early shallow trophoblastic invasion with poor placental and fetal growth restrictions; on the other side are HDPs resulting from maternal “cardiovascular and metabolic risk factors for endothelial dysfunction” [10], both leading to oxidative stress [11].

This study, reported in this Special Issue [12], was designed to investigate the placenta histology and assess if and how it differed between cases of HDP with fetal growth restriction (FGR) and cases of HDP with normally grown fetuses.

In the first trimester, poor placental development and fetal nutrition restriction were frequently associated with maternal hypertension. Eventually, severe placental oxidative stress proved to be a condition associated with a placental pathology with a highly significant prevalence of maternal vascular malperfusion, a condition derived from poor early placental development. The other HDP phenotype associated with “cardiovascular and metabolic risk factors” presented a significant association with an increase in immature terminal villi as a possible result of late hypoxic intervillous space. The decidual acute atherosis observed in these cases and the immature terminal villi link these findings to the other facet of the accelerated, pro-inflammatory placental environment which, in addition to maternal low-grade inflammation from metabolic syndrome, evolves into late-preterm or term HDP. Nutritional and nutraceutical preventive interventions, as discussed in other papers of this Special Issue, could greatly aid in preventing this HDP phenotype.

The SARS-CoV-2 pandemic challenged the immune systems of millions of people worldwide with a devastating outcome. Prior to the development and diffusion of RNA vaccines, immune defenses were overcome, especially in the elderly and in people with obesity. Women of reproductive age were less affected than males, yet pregnancy, and its physiological placental low-grade inflammation in the third trimester, was a biological target for this virus. In countries in which the virus first raged, such as northern Italy, this virus infected up to one in ten pregnant women in spite of the more strict preventive measures adopted by this population [13].

This study, performed in a high-risk maternity ward in Lombardy, hypothesized that glycation and AGE-RAGE oxidative stress in SARS-CoV-2-affected pregnancy might play a role in the severity of COVID-19 syndrome. In fact, this study proved that the methylglyoxal and glycated albumin levels in infected pregnant patients were significantly higher than those in the negative control subjects and that positive pregnant patients who suffered from moderate to severe COVID-19 syndrome had higher values of glycated albumin than those who were infected and presented with mild or no symptoms. On one hand, these findings agreed with the biology of the viral spike protein, which is shielded by glycation and therefore shows a higher affinity for ACE receptors. On the other hand, the findings also proved a connection between the oxidative stress caused by glycation and the severity of COVID-19 in pregnancy.

This Special Issue concludes its overview of possible insights into the role of oxidative stress and pregnancy with a look at the epigenetic profile of obese pregnant women compared to healthy controls. The methylation of miRNA and DNA was analyzed from the saliva of obese and healthy pregnant women after excluding periodontal diseases.

This methodology found that the saliva miRNAs demonstrated significant differences between obese and normal mothers, “involving fatty acids biosynthesis and metabolism, Extracellular Matrix (ECM)–Receptor interaction, and lysine degradation”. Abnormal fatty acid biosynthesis can damage cells and the membranes of organelles, leading to the excessive production of ROS and oxidative stress. Interesting results were also observed for the DNA methylation of transforming growth factor-beta 1 (TGF-Beta1) in the same easily accessible biologic fluid: saliva. TGF-Beta1 is a key cytokine in obesity and insulin resistance [14]. TGF-Beta1 methylation levels were significantly decreased in the saliva of obese mothers vs. lean mothers, underlining the association of obesity in pregnancy with the higher production of TFG-Beta1 cytokines. A similarly reduced methylation was observed for the SOCS3 (suppressor of cytokine signaling 3) gene in the saliva of obese women compared to normal women. SOCS3 is associated with inflammation and insulin resistance. In pregnancy, the possible participation of SOCS3 in the attenuation of leptin and insulin signaling has been recently reported [15]. The findings reported by this original work introduce us to a promising new tool for understanding the complex pathways that regulate inflammation and the possible improvement of our diagnostic skills.
